# Investigating the biodiversity conservation capability of technological innovation and FinTech

**DOI:** 10.1016/j.heliyon.2024.e40683

**Published:** 2024-11-26

**Authors:** Shayan Khan Kakar, Jing Wang, Noman Arshed, Tran Thi Le Hien, Nazir Muhammad Abdullahi

**Affiliations:** a College of Economics and Management, Tarim University, AlaEr, Xinjiang, China; b College of Economics and Management, Northwest A&F University, 3 Taicheng Road, Yangling District, Xianyang, Shaanxi, China; c Department of Business Analystics, Sunway Business School, Sunway University, Kuala Lampur, Malaysia; d Faculty of Finance and Accounting, Ho Chi Minh City University of Industry and Trade, Viet Nam; e School of Rural Technology and Entrepreneurship Development, Kano State Polytechnic, Kano, Nigeria

**Keywords:** Biodiversity, Load capacity curve hypothesis, Dynamic panel analysis, FinTech, Technological innovation

## Abstract

Human activities, primarily economic growth, and technological innovation, threaten global biodiversity. This study utilizes 22-year panel data from 87 developing countries and a novel cross-sectional heterogeneous factor analysis-based financial technology index to investigate how economic growth, renewable energy consumption, technological innovation, natural resources, and financial technology affect biodiversity. To account for cross-sectional dependency, this study employed a Panel Autoregressive Distributive Lagged with Pooled Mean Group specifications within the Driscoll and Kraay standard error estimator. The findings revealed that the log of Gross Domestic Product (GDP) had an inverted U-shaped effect. Moreover, economic growth, renewable energy, and FinTech can improve biodiversity conservation. Traditionally, technological innovation and unregulated resource exploitation have posed threats to biodiversity. This study focused on responsible economic development and practical solutions to biodiversity threats posed by technological innovation and unrestrained resource use. FinTech can promote sustainable behaviors and divert funds from ecosystem-harming projects to biodiversity-friendly ones. Innovative financial instruments enable stakeholders to balance nature. This study demonstrates that FinTech, renewable energy, and responsible economic growth can help reverse biodiversity loss. We provide the policy implications of our research.

## Graphical abstract

**Figure:**
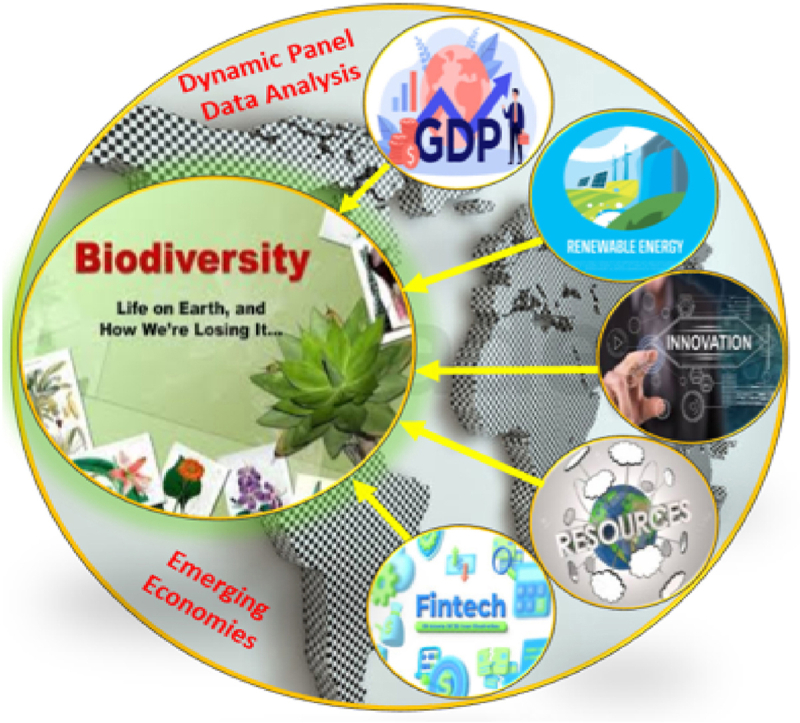

## 1 Introduction

Global biodiversity is rapidly disappearing, threatening future generations [1]. Natural wetlands have experienced a 35 % decline since 1970, while natural forests, covering an area larger than the United Kingdom, have degraded 6.5 billion hectares per year from 2010 to 2015. Since 1970, a steady decline has contributed to the extinction of 60 % of vertebrate populations and the discoloration of more than 30 % of coral reefs [2]. Numerous factors, such as unsustainable exploitation of natural resources, pollution, and land use changes [2], are conduits affecting biodiversity. Despite global attempts to mitigate these risks, biodiversity loss persists [1]. The [2] estimates the annual worth of biodiversity services, such as flood protection, crop pollination, carbon sequestration, and water purification, is between 125 and 140 trillion USD. This value exceeds global GDP by 150 %. Land use and land change (LULC) have increased biodiversity costs to 6–11 trillion USD. Governments must identify socioeconomic indicators of these biodiversity threats [2] as a crucial step toward achieving sustainable development goals.

The socioeconomic context is critical for identifying, addressing, and mitigating biodiversity losses. Human well-being and development depend on urgent remedial measures to address biodiversity deterioration [3]. Escobar [4] stated that exploring the role of economic growth in biodiversity can help reduce biodiversity losses. Empirical studies have examined forestry, infrastructure, and pollution as economic indicators against species endangerment and biodiversity loss [5]. Within this context, the introduction of invasive species threatens native species. Further, there is a coupling between economic growth and greenhouse gas emissions (GHGEs), which rely on fossil fuels [6]. Trade and economic activity are growing, increasing the ecological footprint [7].

The reliance on fossil fuels starts a downward spiral of environmental degradation [8]. Fossil fuels, once the primary driver of global economic growth, are now associated with environmental degradation and biodiversity loss due to growing atmospheric pollution and global biodiversity trends [9]. Renewable energy, which comprised 19.1 % of global energy consumption in 2013, might meet humanity's energy needs [10]. Clark [11] highlights that renewable energy policies spread from 48 nations in 2004 to 164 in 2014. Europe's ambitious target is to use 20 % renewable energy by 2020 [12]. Waste discharges exceeding environmental thresholds have prompted the use of renewable resources (L [13]). Renewable Energy Consumption (REC) minimizes pollution, conserves biodiversity, and promotes sustainable development. Sustainable renewable energy is less harmful to the environment [14]. This source is more essential than fossil fuels, decoupling economic growth and environmental impact [15]. Air pollution from fossil fuel combustion hinders long-term growth and reduces Earth's carrying capacity and biodiversity [16]. Consequently, the hunt for alternative green energy resources has gained momentum as economies recognize the issues with fossil fuels [17]. Researchers and environmentalists have called for green and renewable energy to address fossil fuel-caused biodiversity loss and the energy security dilemma [9].

Renewable Energy Consumption (REC) reduces greenhouse gas (GHG) emissions and climate change [18]; [null]. Several studies suggest that renewable energy preserves natural resources. Tan et al. [19] found that renewable energy preserves natural resources, while [20] found that it harms the environment, particularly natural resources. Renewable energy has environmental and resource implications. Solar energy depletes land, water, and habitat, whereas wind energy projects deforest and transform landscapes [21]. Over 2 billion people worldwide suffer from land degradation, which is expected to worsen due to climate change and human activity [22,23]. They predicted climate change and human activity will worsen land degradation, especially in drier regions. The latest global desertification atlas predicts the destruction of over 90 % of Earth's land by 2050. The United Nations and other international organizations have helped heal ecosystems. In 1977, UNESCO, WMO, and FAO issued the first global desertification map. In 1990, UNEP and ISRIC conducted a study on human-caused soil degradation. They helped pass the UNCCD in Paris on June 7, 1994 [24]. The 2012 UN Conference on Sustainable Development in Rio de Janeiro highlighted the need for governments, organizations, and individuals to collaborate to combat land degradation [25]. In early March 2019, the UN General Assembly designated 2021–2030 as the decade of ecosystem restoration. (Aronson & Alexander, 2013) advised land restoration to combat climate change, boost food security, and protect water and biodiversity.

Conversely, economic have been activities associated with driving energy consumption and GHGEs. Unfortunately, the UN Output Gap Report 2021 warns that current output projections violate the Paris Agreement constraints [26]. Higher economic growth increases energy demands, pollution, and biodiversity loss [27,28]. The global economy's interdependence due to globalization and competition necessitates the utilization of low-cost labor from developing countries to boost productivity and production. Eco-friendly technologies can also assist underdeveloped nations [29]. Pursuing economic growth while curbing environmental degradation is not a novel concept; harnessing biodiversity to promote green growth and enhanced environmental quality is a relatively recent development [28].

Technological innovation is essential for environmental protection and sustainable development, especially biodiversity preservation [null]. Governments in all economies must reduce pollution and achieve sustainable development to address biodiversity challenges. Technological innovation can balance economic growth and ecological protection when addressing biodiversity-related environmental issues [30]. Technological advancements often lead to the development of eco-friendly products, methods, and procedures [31]. Supporting the advancement of technology can stimulate biodiversity and protect the environment [32]. Moreover [33], asserts that technological innovation holds significant potential as a contemporary strategy to halt biodiversity loss and alleviate climate change harm [34]. contend that factors such as increasing reliance on fossil fuels, economic growth, financial system advancements, and urbanization contribute to environmental pollution and biodiversity loss. Utilizing renewable energy sources enhances economic development through technical advancements while reducing adverse environmental effects [35].

Social and economic progress requires effective and responsible use of natural resources ([19]; Y [36]). Nevertheless, rapid industrialization and population growth have increased the demand for these resources, resulting in reckless depletion and damage to the ecosystem and biodiversity [37]. Sustainable resource management and laws are essential for long-term ecological equilibrium [19]. Exports and investment-led growth have depleted natural resources and harmed the ecosystem [7]. A thorough accounting of economic activity and its implications is required [38]. This can help in balancing ecological and human sustainability.

Integrating financial systems and technology (FinTech) helps form digital accountability for ecological degradation using financial instruments. It promotes efficient resource utilization and demand management (Keys et al., 2019; Mi & Coffman, 2019). Integrating FinTech with online platforms can help reduce pollution from consumption and production (Makov et al., 2020; Zhou et al., 2020). The transformation towards mobile transactions has helped rural development and digitize the agri-business sector [39]. FinTech can assist in identifying and evaluating environmentally sustainable practices in the banking industry, making environmental management easier (W [40]). As a result, FinTech has the potential to act as a green technology [37]. Experts credit green technological innovation for enhancing environmental stewardship, mitigating environmental degradation, and promoting sustainable and balanced economic development. Innovation mitigates biodiversity loss by introducing new products, methods, and procedures [null]; [31].

This study adds to our understanding of the relationship between economic growth and biodiversity globally. Since previous empirical research has needed more definitive findings, understanding the relationship between economic growth and biodiversity poses a formidable challenge, especially globally. The concept of the Environmental Kuznets curve, which links to biodiversity, influences the relationship between economic growth and biodiversity through renewable energy, technological innovation, natural resources, and financial technology. This study aims to investigate the nature of the nonlinear relationship between economic growth and biodiversity conservation, using squared GDP as the independent variable. The importance of biodiversity grows in tandem with economic growth. However, the impact on biodiversity declines after reaching a certain threshold.

Furthermore, the study investigates the impact of renewable energy, technological innovation, natural resources, and financial technology on biodiversity. This study investigates the effect of these factors on biodiversity in a novel way. Furthermore, this study employs a robust econometrics technique, called panel ARDL – with PMG specification within the Driscoll-Kraay standard error (DKSE) estimator to address the potential issues of heterogeneity, cross-sectional dependence, autocorrelation, and heteroscedasticity in the panel data collected from the 87 economies. This methodology improves effectiveness and yields dependable outcomes.

The study is structured as follows: Section 2 organizes a comprehensive literature review into various sections that pertain to the study variables. Section 3 describes the methodology employed in the study, including the theoretical framework. Section 4 presents and discusses the study findings. The paper's final section offers a conclusion, summarizes the study's key findings, and provides policy implications derived from the research.

## 2 Literature review

### 2.1 Relationship between economic growth and biodiversity

Economic research into the underlying causes of species extinction on a global scale is still in its infancy. Most studies on this topic concentrate on determining how economic growth impacts biodiversity. Beck and Nesmith [41] reported 2.5 billion people in 1950 and 9 trillion USD in GDP in 2011. World prosperity raised the average yearly income to $3300 PPP, which is high by historical standards. Since 1950, global poverty has fallen from 60 % to 10 % (1.90 USD/day) [42]. In 2011, the global output of goods and services exceeded 120 trillion dollars (PPP), the population exceeded 7.7 billion, and the GDP per capita exceeded $16000. A 13-fold rise in economic activity over 70 years is incredible. Although successful, the environment has degraded. Researchers such as Kerr and Currie [43], Naidoo and Adamowicz [44], Asafu-Adjaye [45], Dietz and Adger [46], McPherson and Nieswiadomy [47], and Halkos and Tzeremes [48] have proven this tendency. The International Union for the Conservation of Nature's (IUCN) red list helps researchers count endangered species. Dietz and Adger [46] are the only studies that use time series data to track biodiversity changes depending on forest coverage, based on the hypothesized relationship between forest area and biodiversity. Gross domestic product (GDP) per capita is a standard economic growth indicator in these studies ([28]; X [49]) test the Environmental Kuznets curve EKC hypotheses using quadratic or cubic GDP per capita components to determine the non-linear relationship between biodiversity and economic growth.

According to Naidoo and Adamowicz [44] and McPherson and Nieswiadomy [47], the inverted-shaped curve for amphibians and reptiles demonstrates that economic growth harms them. Exclusive economic growth studies provide conflicting and mixed results. Kerr and Currie [43] and Dietz and Adger [46] suggest that higher economic growth promotes biodiversity. However, Asafu-Adjaye [45] discovered the inverse relationship between economic growth and biodiversity. Halkos and Tzeremes [48] developed a biodiversity performance indicator that links country environmental variables to known and vulnerable species. As GDP per capita rises, biodiversity performance deteriorates. An ecological study links biodiversity loss to climate change, habitat destruction, fragmentation, and non-Indigenous species (NIS). Researchers have been studying how climate affects biodiversity since the early 18th century. Rohde [50] and others investigated climate variables such as temperature and precipitation, which increase biodiversity. The biggest risks to biodiversity are habitat loss and degradation, mostly induced by land use changes. Luck [51] suggests that agribusiness, urbanization, water development, and forestry affect habitats. Butchart et al. (2010) claim that they are crucial to habitat changes that threaten biodiversity. Clavero et al. [52] and Spear and Chown [53] propose how NIS reduces species richness. Increased predation, competition, or new diseases caused by NIS-borne pests directly and indirectly impact local plants and animals.

Arshed et al. (2023) conducted a study where they examined the quadratic relationship between GDP, biodiversity, and ecological vitality across 57 countries. The researchers utilized the biodiversity index from the Environmental Performance Index. The study determined that GDP has a U-shaped effect on biodiversity, as evidenced by the panel ARDL model. The results confirmed the premise of the load capacity curve hypothesis (LCC). In a study conducted by Arshed et al. [9], it was shown that when biodiversity is at low levels, GDP has a detrimental impact. These findings were observed across 66 nations.

### 2.2 Relationship between renewable energy consumption (REC) and biodiversity

According to the (IPCC 2013), fossil fuels are the driving force behind climate change. In 2013, the World Health Organization (WHO) identified fossil fuels as a major cause of air pollution. In addition to biodiversity loss, fossil fuel extraction promotes human activities that harm, degrade, and fragment ecosystems [54]. International treaties handle these two significant environmental challenges. These agreements establish medium-term policy objectives and priorities. The Kyoto Protocol and the 2009 Copenhagen Accord were UNFCCC-supervised climate change mitigation efforts. The Convention on Biological Diversity established the Aichi Goals 2010 to conserve biodiversity globally. Climate change and biodiversity loss pose significant environmental issues. Despite the need for international cooperation to address these issues, governments usually act independently [55].

International carbon credit markets can help facilitate global climate change collaboration ([55]; REN21, 2016). Bioenergy production and trade, a major renewable energy source, have grown [56]. Tropical bioenergy is economically viable and employed by industrialized nations with high energy demands and a desire to reduce emissions. Wind and solar, other fast-growing renewable energy sources, are limited to their generation locations [55]. Wind and solar energy may be less likely to benefit from a globally coordinated energy policy. However, with political determination, global cooperation can protect biodiversity. Pouzols et al. [57] suggested expanding the global Protected Area (PA) network. This refers to expanding protected areas and helping conserve global biodiversity. Climate change and biodiversity loss require international collaboration with strategies varying by environment. International carbon credit markets can combat climate change, but expanding the global network of protected areas can help preserve biodiversity through collaborative efforts. Researchers have examined renewable energy and its environmental impact using different methodologies. Joof et al. [58] examined the insurance market, climate change, and biodiversity in BRICS nations. The author finds that renewable energy boosts biodiversity using NARDL. Gasparatos et al. [59] investigated how direct and indirect renewable energy affect biodiversity. Balsalobre Lorente et al. (2023) state that fossil fuels and other polluting energy sources drive mining, infrastructure development, transportation, and fossil fuel combustion, which harm biodiversity.

To better understand energy use, researchers looked at ecological markers. Danish et al. [60] examined how energy and resource utilization impact OECD carbon emissions and environmental footprint. Renewable energy sources help the environment, whereas non-renewable energy sources hurt it. The environmental Kuznets Curve (EKC) suggests that environmental degradation worsens before improving in developing nations. Haldar and Sethi [61] analyzed national ecological circumstances using the MG, AMG, and DCCE models. The study concluded that renewable energy enhances environmental quality, whereas non-renewable energy degrades it. Xue et al. [62] found that renewable energy enhances ecological well-being in Bangladesh, India, Pakistan, and Sri Lanka. Further studies by Usman and Radulescu [63], and Sharma et al. [64] demonstrate that renewable energy enhances environmental quality, which affects biodiversity [16]. In their study, Arshed et al. (2023) employed renewable energy sources to assess their influence on biodiversity. They found that renewable energy has an insignificant influence on biodiversity, and carbon reduction is essential to protecting it. Due to the scale effect, which causes growth and strains natural resources, Arshed et al. [9] warn that renewable energy could endanger biodiversity. This suggests exploring renewable energy.

### 2.3 Relationship between technological innovation and biodiversity

Government actions aimed at reducing carbon emissions, which are a major cause of climate change, have led to the development of technology and economic growth. Technological innovation plays a role in diminishing the environmental consequences of economic activities. Researchers used patent data to investigate technological advancements, as demonstrated by several studies (J. [[65], [66], [67], [68], [69]]). Research and Development R&D spending can serve as an additional indication of innovation [70]. Wydra [71] and Urbaniec et al. [72] found that resident and non-resident patent applications reliably indicate technological innovation. Technological innovation entails developing new products and methods for conserving energy, reducing waste, mitigating pollution, and improving environmental management [72]. assert that neoclassical theorists claim technological innovation is pivotal in attaining environmental sustainability. Technological innovation fosters economic growth while simultaneously protecting the environment, a crucial aspect for achieving sustainable development in the long run [66]. Recent studies indicate that technological innovation enhances corporate competitiveness, sustainability, and growth ([73]; C.-T. [[74], [75], [76]]). Because it saves energy and resources, governments can use technology to solve environmental problems and accomplish sustainable development goals.

According to a recent study, economic progress and government intervention promote technical innovation. Economic growth depends on several things. Economic growth drives innovation because a larger market can afford to invest more in technology. In Singapore, Meirun et al. [77] used the bootstrap autoregressive-distributive lag (BARDL) approach to find a short- and long-term positive relationship between environmental quality and technological innovation. Empirical studies of the Porter hypothesis are inconsistent and integrated [78]. Numerous studies have found that environmental constraints encourage technological innovation. Such regulations drive businesses to find cheaper compliance solutions. Environmental regulations, such as taxes, may hinder technological innovation by raising operating costs [66]. Hottenrott & Rexhäuser [79] project that environmental regulations and technological innovation are nonlinear [80]. According to Ai et al. [81], there is a U-shaped effect between environmental pollution and technological innovation, showing that regulation may initially inhibit innovation but eventually encourage it. Wang et al. [82] confirmed an insignificant effect. When connecting to biodiversity, the influence of environmental regulations on technological innovation becomes ambiguous and complex [16].

### 2.4 Relationship between natural resources and biodiversity

Climate change presents a growing and significant risk to natural ecosystems and biodiversity. This phenomenon profoundly impacts ecosystems, affecting their composition, functioning, and societal benefits [83]. Human civilizations can improve their readiness and flexibility by understanding the nature and extent of ecological reactions. Climate change impacts on current and future ecosystems must be evaluated regularly to establish and modify natural resource management strategies and review implementation plans [84]. The Global Change Research Act mandates the United States to conduct a National Climate Assessment (NCA) every four years. It examines the existing and projected consequences of climate change on all sectors and regions in the country [85]. For the most up-to-date assessment of how climate change affects ecosystems, biodiversity, and the services they provide in the United States (US), we refer to the fourth National Climate Assessment (NCA4) volume 2 in Chapter 4, which addresses natural resources such as ecosystems and biodiversity [85].

Earlier researchers focused on mineral-exporting nations and used carbon emissions as a metric of environmental quality, with the underlying premise being that increased carbon emissions lead to environmental issues and vice versa Pachiyappan et al. [86]. However, this study measures environmental quality in terms of biodiversity. Among these studies, Hussain et al. [87] analyze data from China. China This exports a significant amount of minerals, and research reveals that while the consumption of natural resources boosted the country's economy, it also contributes to environmental issues by increasing its carbon emissions. Their findings also revealed that financial growth had the opposite impact, lowering carbon emissions in China, whereas inflationary pressures and increased population density contributed to higher carbon emissions. Their result also supports the environmental Kuznets curve (EKC) theory, which states that carbon emissions increase at the outset of a country's economic growth before decreasing as the economy develops. However, Lee et al. [88] and Shen et al. [89], using provincial data from China, found that urbanization harms environmental quality. However, these effects will reverse when the Chinese financial industry develops sufficiently. To reduce provincial carbon emissions, the author of the previous study stressed the significance of growing green investment. In Japan, a significant mineral resource exporter, Shahbaz et al. [90] showed that economic growth policies initially reduce carbon emissions. They concluded that energy consumption and globalization boost Japan's annual short-term emissions. Salari et al. [91] examined the link between annual carbon emissions, energy consumption, and economic growth in the US mineral-exporting economy. Their findings showed that greater energy use in the US increases carbon emissions over time. Their analysis also supported the concepts of the environmental Kuznets curve (EKC).

Ul-Durar et al. [37] examined the impact of natural resource rents on biodiversity and environmental performance in Asian countries. They determined that excessive use of natural resources would typically result in biodiversity depreciation. The findings align with the research conducted by Iqbal et al. [35] on nations abundant in resources.

### 2.5 Relationship between financial technology (FinTech) and biodiversity

Fintech manages personal and business finances with artificial intelligence, machine learning, digital assistants, and mobile apps. Sustainable development models will produce $12 billion annually by 2030 [92]. Fintech, which uses cutting-edge technology such as big data analytics, bitcoin, and other new platforms, assists businesses in reducing waste and steering shareholders toward environmentally friendly products. Muganyi et al. [93] studied how eco-friendly regulations reduced carbon emissions in China from 2011 to 2018. They discovered that, by utilizing the semi-parametric difference-in-differences (SDID) technique, Chinese FinTech companies significantly reduced their carbon emissions. The growth of FinTech has also stimulated environmental investment. Since China may lead green finance network rules, the government should boost FinTech growth and banks' green loan capacity [94]. The author uses SDID to demonstrate how India's green funds and environmental laws have reduced industry carbon emissions. FinTech enables businesses to minimize carbon emissions, manage portfolios, and conserve energy [95].

Chueca Vergara and Ferruz Agudo (2021) conducted a literature study and case studies focusing on financial technology and ecological responsibility. They concentrated on specific applications of advanced technology to drive green finance and FinTech growth. Correct FinTech development may prevent environmental deterioration and improve ecological integrity. Tao et al. [96] used 2Sls and GMM calculations to obtain accurate results, even after accounting for FinTech growth endogeneity. Croutzet and Dabbous [97] argue that FinTech drives OECD green energy adoption due to concerns about energy security, fluctuating oil costs, and a desire to reduce carbon emissions. Fintech impacts long-term energy expenditure, savings, and investment. FinTech is helping to create a sustainable ecosystem and fulfill customers' environmental concerns. The BRICS nations are also considering FinTech's impact on sustainable development. Future research will examine green computing, FinTech characteristics, ESG reforms, and FinTech's environmental advantages. Udeagha and Muchapondwa [98] found that new financial technologies may help BRICS nations reduce carbon emissions. They urged expanding green funding and finding innovative energy solutions to reduce carbon emissions. However, economic growth and resource extraction harm the ecosystems of the BRICS nations.

Turning our attention to the United States, Saqib et al. [99] suggested that FinTech can improve environmental quality by reducing ecological footprints today and in the future. They also found that filthy energy increases environmental footprints whereas clean energy decreases them. Additionally, the study supported the EKC hypothesis in the US. Firdousi et al. [100] investigated data from 26 developing nations, including major mineral exporters, to show that FinTech development reduces carbon emissions and promotes renewable energy consumption. Lu et al. (2023) used BRICS data to investigate long-term carbon emission reduction in FinTech. In conclusion, the literature illuminates the complex relationship between financial technology (FinTech) and environmental quality, particularly how FinTech development impacts environmental outcomes in different countries.

A study by Ul-Durar et al. [37] analyzed the impact of FinTech on biodiversity from the mineral management perspective of One Belt and One Road countries in Asia. The results showed that a rise in FinTech led to improved biodiversity and environmental performance. Another study by Iqbal et al. [35] assessed the role of FinTech on biodiversity in resource-rich countries using second-generation panel quantile regression. This study also concluded that FinTech had a positive effect on biodiversity.

### 2.6 Research gap

There have been several studies that empirically linked socioeconomic activities with biodiversity. This study fills the gap in several major ways. Firstly, empirical studies often mix innovation and financial technology, which can have different effects. Secondly, researchers rarely discuss the proposed socioeconomic indicators for biodiversity together. Thirdly, this study has used cross-section-specific factor analysis to get around the problem that comes up with factor analysis for panel data when co-variance can be different for each cross-section [37]. Lastly, this study integrated the panel ARDL specification with the DK estimator to form a novel set of equations that addresses all major issues in the panel data.

## 3 Methodology

This study examines the complex relationship between economic growth, renewable energy consumption, technological innovations, natural resources, Fintech, and biodiversity. The data is acquired from World Development Indicators, the International Monetary Fund, and the Yale University Environmental Performance Index. This study uses data from 87 economies to investigate these associations with biodiversity as the dependent variable. Zhang et al. [101] examined FinTech's biodiversity impact in 23 economies. Ali et al. [18] also used bootstrapping ARDL to examine how technological innovation, natural resources, and renewable energy affect the United States' ecological footprint of the United States. Gasparatos et al. [59] found that renewable energy has a major impact on Southeast Asian biodiversity [102]. tested the Environmental Kuznets Curve (EKC) hypotheses using square and linear GDP per capita to explore the link between GDP, environmental quality, and biodiversity. The curvilinear relationship for the relative growth-biodiversity decoupling effect is estimated using GDP per capita squared.

### 3.1 Data construction and sources

This study used 2000–2021 global panel data from 87 economies, as listed in Appendix 1. The dataset is sourced from the World Development Indicator (WDI), International Monetary Fund (IMF), and Environmental Performance Index by Yale University. Table 1 details the dependent and independent variables, unit of measurement, and data source. Furthermore, the dataset comprises 1845 observations, facilitating a robust analysis of the research objectives. This study investigates the impact on biodiversity, which is an index of seven indicators: the Biodiversity Habitat Index, Marine Protected Areas, Protected Areas Representativeness Index, Species Protection Index, Species Habitat Index, Terrestrial Biome Protection (National), and Terrestrial Biome Protection (Global). The data for these indicators can be found in the publication by Ref. [103]. Arshed et al. (2023) have empirically adapted this indicator of the Environmental Performance Index. This study uses the Financial Development Index, Mobile Subscriptions, and Internet usage to calculate the FinTech index as proposed by Ref. [37,95]

**Table 1:** Detailed description of the variables used in the study.

Variables	Abbreviations	Measurement unit of variables	Sources
Biodiversity	Bio	Sub Index of Environmental Performance Index	Environmental performance index Yale University [103] used by Ul-Durar et al. [37], Arshed et al. [9] and Iqbal et al. [35]
Economic Growth	GDP	GDP per capita (current US$)	WDI
Renewable Energy	REN	% of total final energy consumption	WDI
Technological Innovation	IN	Patent application resident, non-resident	WDI
Natural Resource Rent	NRR	Total natural resource rent % of GDP	WDI
Financial Technology	FinTech	Index of FDI, MPS and IU provided below	Method adopted from Ul-Durar et al. [37]. Index adopted from Iqbal et al. [35] and Kakar et al. [95]
Financial Development Index	FDI	Financial development index	IMF
Mobile phone subscription	MPS	Mobile cellular subscriptions (per 100 people)	WDI
Internet users	IU	Individuals using the internet (% of population)	WDI

### 3.2 Theoretical framework

**Fig. 1:**
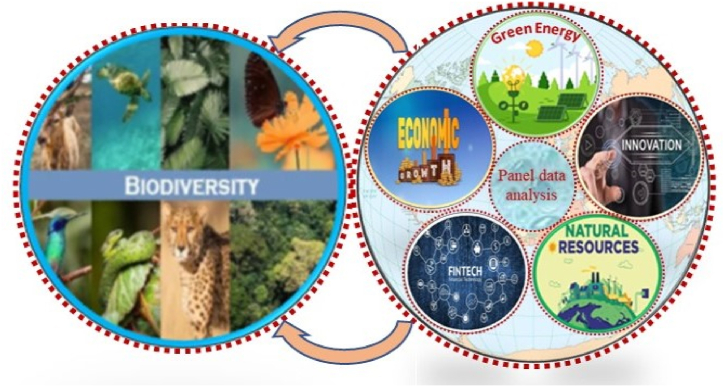
Theoretical framework.

Referring to the provided theoretical framework in Fig. 1, we can use a panel ARDL model to examine the intricate relationship between biodiversity, economic growth, renewable energy, technological innovation, natural resources, and FinTech. Empirically, studies have compared environmental quality, specifically carbon emissions, to GDP and GDP squared to form the Environmental Kuznets Curve (EKC) [104,105]. We use GDP in standard units to verify the absolute delinking between the environment and GDP, while the log of GDP is used to check relative delinking. Further, this study extends the model by assessing the EKC hypothesis for the case of biodiversity adaptation [9,28,106]. Further controlling factors discussed in the literature are added to assess their potential to shift the EKC upward or downward. Positive affecting factors lead to an upward shift in the EKC, thereby increasing biodiversity at any given GDP value, and vice versa.

### 3.3 Equations

The model below has been constructed to assess the impact of economic growth, renewable energy, technological innovation, natural resources, and FinTech on biodiversity at the national level. Literature has pointed out that the quadratic transformation employs the lGDP and its EKC or LCC effect.

(1) $${BIOD}_{it}=\alpha_{1}+\alpha_{2}{lGDP}_{it}+\alpha_{3}{lGDP}_{it}^{2}+\alpha_{4}{REN}_{it}+\alpha_{5}{lIN}_{it}+\alpha_{6}{NRR}_{it}+\alpha_{7}{FINTECH}_{it}+e_{it}$$

(2) $${\Delta BIOD}_{it}=\alpha_{1}+\alpha_{2}\Delta {lGDP}_{it}+\alpha_{3}\Delta {lGDP}_{it}^{2}+\alpha_{4}\Delta {REN}_{it}+\alpha_{5}\Delta {lIN}_{it}+\alpha_{6}\Delta {NRR}_{it}+\alpha_{7}\Delta {FINTECH}_{it}+{\delta_{8}e}_{it-1}+\mu_{it}$$

Here, BIOD = Biodiversity Index, lGDP = log of GDP, REN = Renewable energy % of the total, lIN = log of innovations, NRR = Natural resource rents % of GDP, FINTECH = Fintech index using heterogeneous factor analysis. The long-run estimates come from Equation (1), and the residuals are used in the first difference Equation (2) to develop the 2-step ECM approach discussed by Ref. [107]. This method estimates the restricted form ARDL equation, which absorbs the first lag of BIOD to form ΔBIOD [9,37,108].

### 3.4 Model estimation

#### 3.4.1 Heterogeneous factor analysis

This study has constituted the FinTech index using the heterogeneous factor analysis method, allowing for cross-sectional differences between previously described data sets [109,110]. Cross-sectional KMO [111] and Bartlett's [112] tests validated this index. These statistics, along with the country-specific factor loading values, highlight the characteristics of the constituted index. A previous study by Ul-Durar et al. [37] used this method to generate a more efficient index that accounts for the differences in data across cross-sections.

#### 3.4.2 Panel unit root, cross-sectional dependency, and panel cointegration test

Since the number of years per country is long enough, this study resorts to dynamic panel data models to achieve its objectives while addressing non-stationary variables. The panel ARDL model can handle a mixed order of integrated variables [113], so unit root tests were used to ensure that none of the variables is I (2). This study also used second-generation panel unit root tests developed by Pesaran [114]. This study also used a cross-sectional dependence test to confirm the cross-sectional dependency in the data [115]. This study confirmed the presence of long-run relations using the second-generation panel cointegration test [116].

#### 3.4.3 2-Step ECM with Driscoll Kraay standard error regression

The Driscoll and Kraay (DK) standard error estimator, developed by Ref. [117], has several benefits for econometric analysis, especially panel data modeling. This panel data model can conventionally handle unobserved heterogeneity, while it is instrumental in making standard errors robust to cross-sectional dependence [118]. This makes this estimator a second-generation panel data model.

This study uses this estimator to form an ECM equation, confirming the validity of long-run estimation through panel cointegration (section 3.4.2) and the panel unit root test on the long-run equation residuals. The confirmation of stationarity of residuals confirms the absence of spuriousness in estimates.

#### 3.4.4 Pooled mean group specification

The 2-Step ECM method [107] then uses the long-run relation to form a restricted ARDL equation, made possible by the presence of cointegration. Blackburne and Frank [119] also used this method to form the Panel ARDL model within the OLS regression estimator. Using Pesaran and Smith's [113] specification, Blackburne and Frank [119] discuss the panel ARDL model's three variants and provide their equations. This study adopts the pooled mean group (PMG) specification, which assumes that the long run is homogeneous across cross-sections and the short run is heterogeneous. A study like [28] employed this PMG specification in panel data setup. This study incorporates this specification into the DK estimator to enhance the robustness of cross-sectional dependence.

#### 3.4.5 Panel granger causality test

This study utilized the granger causality test to investigate the causal relationship between variables in panel data. These tests extend the conventional Granger causality test, determining if one time series may predict another [120]. Panel data allows you to analyze causality between variables while accounting for individual specific effects and temporal dynamics across numerous cross-sectional units. The null hypothesis of Granger causality: There is no Granger causality between the variables. The alternate hypothesis is that there is Granger causality between the variables. This study used the panel non-causality test [121].

## 4 Results and discussions

### 4.1 Heterogeneous factor analysis

**Fig. 2:**
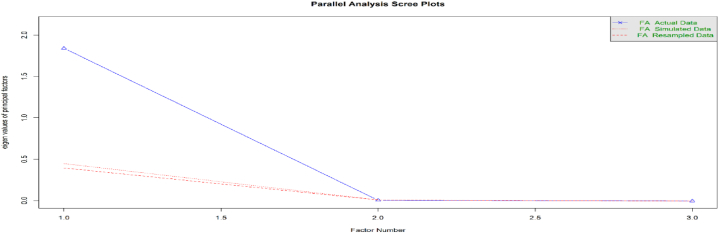
– Screen plot.

**Fig. 3:**
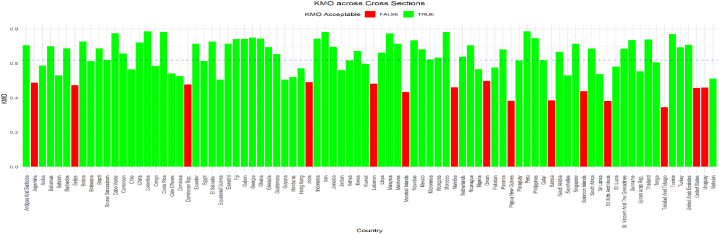
– Country Specific KMO values.

**Fig. 4:**
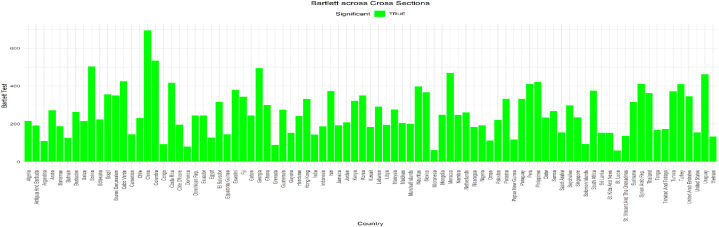
– Country-Specific Bartlett's test.

Since this study uses panel data from 87 nations, the data may exhibit heteroscedasticity. The study used three variables to form the FinTech index. It includes the proportion of the population with access to the internet, the proportion of the population with access to mobile phones, and the proposed financial sector development index [122]. The scree plot in Fig. 2 shows that only one index is suitable for these three variables. The overall sample KMO yielded 0.62, which exceeded the 0.50 threshold, while Fig. 3 shows that most countries had suitable KMO values. However, when the overall KMO exceeds 0.50, we can ignore the few countries with a KMO below 0.50. Fig. 4 shows that the overall and country-specific Bartlett's test is significant, confirming the data's indexability.

**Fig. 5:**
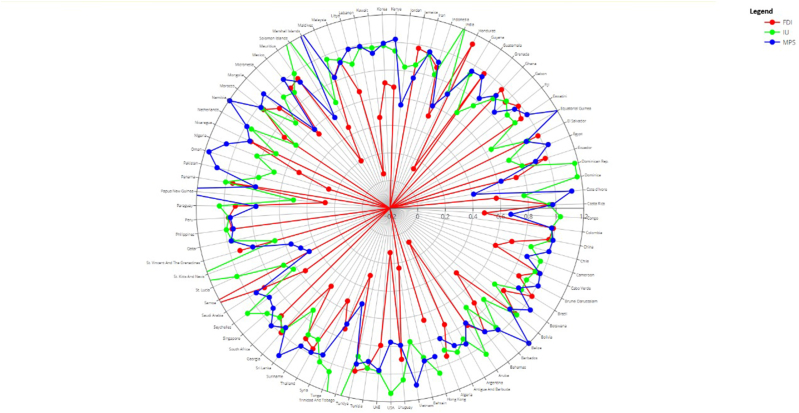
– Country specific factor loading values.

The index for the entire sample showed factor loadings of 0.75 for a Mobile Subscription (Mobs), 0.990 for an internet subscription (inter), and 0.543 for Financial Market Development (FMD), originally the Financial Development Index (FDI). Fig. 5 shows country-specific factor loading values. This helps identify the composition of the index in each country. The spider plot shows the financial market development index loadings that vary most between countries. Since the factor loadings differ from normal factor analysis, which yields one-factor loading for each variable in the sample, cross-sectional heterogeneous factor analysis is necessary.

**Fig. 6:**
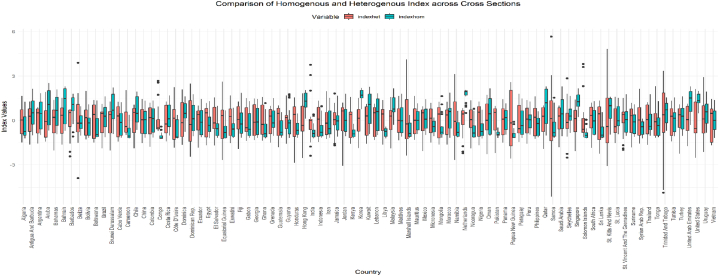
– Country-wise comparison of overall and heterogeneous factor analysis-based indices.

Fig. 6 compares between the standard factor analysis-constituted index and the cross-sectional heterogeneous factor analysis. The indices differ little, except for the fact that FinTech's heterogeneous index (indexhet) is centered at zero for all cross-sections, whereas the standard factor analysis is centered at zero in the overall sample but not country-wise sub-samples, making them comparable across countries.

### 4.2 Descriptive statistics

Table 2 shows the descriptive statistics analysis for selected variables. The mean values of biodiversity, GDP, and technological innovation are greater than their standard deviation, indicating that these variables are overspread. However, the mean value of renewable energy, natural resources, and FinTech is less than the standard deviation, indicating that these variables are under spread. Moreover, the skewness results show both negative and positive skews in the variable. Only GDP exhibits a negative skew, as predicted for a particular nation.

**Table 2:** – Descriptive statistics.

Statistics	BIOD	lGDP	REN	lIN	NRR	FINHET
Observation	1845	1845	1845	1845	1845	1845
Mean	38.307	24.272	24.300	5.627	8.243	−0.000
Median	38.215	24.324	15.710	5.438	2.709	0.172
Std. Dev.	21.758	2.383	24.646	2.668	12.038	1.135
Skewness	0.250	−0.031	1.042	0.743	2.169	−0.130
Kurtosis	2.105	2.631	3.239	3.350	8.214	3.728
JB test	205.740	14.560	189.550	58.530	635.760	25.480
Probability	0.000∗∗∗	0.000∗∗∗	0.000∗∗∗	0.000∗∗∗	0.000∗∗∗	0.000∗∗∗

∗∗∗ significant at 1 %.

**Fig. 7:**
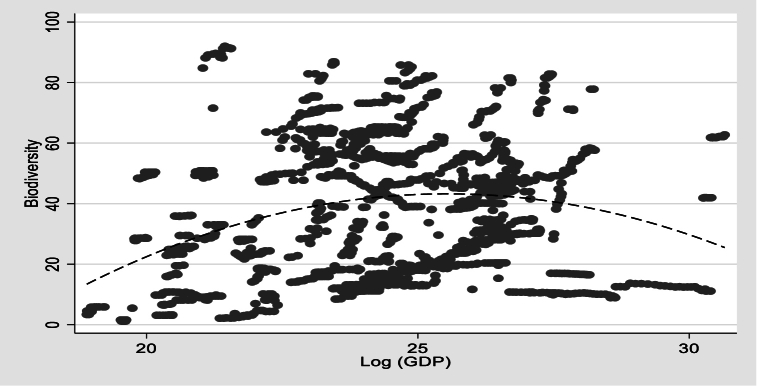
– Non-linear association between lGDP and BIOD.

In contrast, kurtosis shows the issue of outliers, especially in the case of natural resource rents. Fig. 7 shows that there is a nonlinear association between lGDP and BIOD. The quadratic fit shows inverted U-shaped patterns. Initially, biodiversity conservation increases in tandem with economic growth. However, as growth beyond a certain threshold, biodiversity conservation begins to diminish.

### 4.3 Correlation matrix analysis

Table 3 displays the correlation matrix analysis. Here, we can see that lGDP, REN, lIN, and FINTECH are positively associated with biodiversity, while NRR is negatively associated. The comparison of pairwise correlations between independent variables revealed no high correlations, confirming the absence of multicollinearity [123].

**Table 3:** Pairwise correlations.

Variables	BIOD	lGDP	REN	lIN	NRR	FINHET
BIOD	1.00					
lGDP	0.192	1.00				
REN	0.218	−0.012	1.00			
lIN	0.043	0.838	−0.134	1.00		
NRR	−0.025	0.089	−0.074	−0.062	1.00	
FINHET	0.133	0.073	−0.044	0.058	−0.067	1.00

### 4.4 Panel unit root, cross-sectional dependence, and cointegration analysis

Table 4 depicts the second-generation panel unit root test deployment. Other than innovation (IN), all of the variables are insignificant at the level but significant at the first difference, implying that they are I (1) in nature while IN is I (0) in nature. The mixed order of the data demonstrates the use of the Panel ARDL model.

**Table 4:** Panel unit root and cross-sectional dependence test.

Variable	Z [t-bar] (P value)		Cross-sectional dependence
	Level	First difference	CD-Test (P value)
Bio	1.709 (0.95)	−2.413 (0.01)∗∗	112.43 (0.00)∗∗∗
lGDP	−1.267 (0.10)	−7.867 (0.00)∗∗∗	210.84 (0.00)∗∗∗
REN	0.605 (0.73)	−2.025 (0.02)∗∗	26.94 (0.00)∗∗∗
IN	−2.614 (0.00)∗∗∗	–	84.99 (0.00)∗∗∗
NRR	0.759 (0.78)	−4.737 (0.00)∗∗∗	80.57 (0.00)∗∗∗
FinTech	−0.749 (0.23)	−4.962 (0.00)∗∗∗	216.46 (0.00)∗∗∗

∗∗∗ significant at 1 %, ∗∗ significant at5%, ∗ significant at 10 %.

Table 4 also provides the panel cross-sectional dependence test, which confirms that the data is cross-sectionally dependent (cross-sectional auto-correlated). This necessitates the use of second-generation models. The Driscoll and Kraay estimate was used in this study. It is a second-generation model that manually estimates the Panel ARDL model specification with a pooled mean group, thereby creating a second-generation Panel ARDL model. This study is instrumental in integrating the robust cross-sectional dependence estimates with the ARDL specification to create this hybrid model.

Table 5 shows that the second-generation Westerlund panel cointegration test, with an alternative hypothesis involving some cointegration panels, yielded a p-value of 0.038, below the significance level of 0.05, implying that the null hypothesis can be rejected at the 5 % level. The test with all panels cointegrated yielded a p-value of 0.068, which is less than 0.10, thereby rejecting the null hypothesis at the 10 % level. Thus, panel variable cointegration implies a long-run relationship. Murshed [122] used the Westerlund cointegration test to determine the long-term relationship between financial technology (FinTech) and mineral resource environmental issue remediation.

**Table 5:** Panel Cointegration test.

Westerlund	Test statistic	P value
Ratio Test (Some Panels)	−1.7657	0.038∗∗
Variance Ratio Test (All Panels)	−1.484	0.068∗

∗∗ significant at 5 % ∗ significant at 10 %.

### 4.5 Regression with Driscoll Kraay pooled mean group long-run

Table 6 shows Panel ARDL estimates from Driscoll and Kraay's robust regression technique to address common correlation shocks and unobserved heterogeneity in panel data. We are applying this approach to make estimates robust to cross-sectional dependence. Our primary goal is to estimate the regression coefficient while considering observational interdependence across all countries in our data set [101]. We also use a second-generation panel autoregressive distributed lag (PARDL) model with a pooled mean (PMG) specification to investigate the relationship between economic growth, renewable energy consumption, technological innovation, natural resources, financial technology (FinTech), and biodiversity. The unit root tests conducted on the residuals from Equation (1) confirmed stationarity at the level; indicating the presence of a long-run relationship and the absence of spuriousness in Equation (1). The use of DK regression makes estimates robust to dependence, while the use of PMG makes the model robust to heteroscedasticity.

**Table 6:** – Long and short-run estimates of Panel ARDL with PMG using DK estimates.

Variables	Coefficient	P-value	Variables	Coefficient	P-value
Long run estimates			Short run estimates		
lGDP	26.196	0.000∗∗∗	ΔlGDP	3.622	0.720
lGDP^2^	−0.464	0.000∗∗∗	ΔlGDP^2^	−0.077	0.720
REN	0.135	0.000∗∗∗	ΔREN	−0.004	0.850
lIN	−2.018	0.000∗∗∗	ΔlIN	0.040	0.890
NRR	−0.167	0.000∗∗∗	ΔNRR	−0.008	0.480
FINHET	2.332	0.000∗∗∗	ΔFINHET	0.125	0.420
			ecm_-1_	−0.152	0.000∗∗∗
cons	−311.602	0.000∗∗∗	Cons	0.597	0.000∗∗∗
F	1296	0.000∗∗∗	F	28.540	0.000∗∗∗
R^2^	0.1537		R^2^	0.080	
sample	1845		sample	1758	
Countries	87		Countries	87	

∗∗∗ significant at 1 %.

The study's long-run estimates are based on 1845 observations from 87 countries. In the long and short run, the independent variables explain 15.37 % and 0.08 % of the dependent variables, respectively.^1^ The significant F test validates the fit of both the long- and short-run models, thereby confirming the effectiveness of the proposed variables. Since the absence of financial dependent variables and the scarcity of biodiversity studies, developing a model with a 15.37 % explanation is expected in the long run. At the same time, a low short-run R squared depicts that the variables used in the study do not create disturbance in the short-run but are slow to change, and their change is visible in the long-run.

**Fig. 8:**
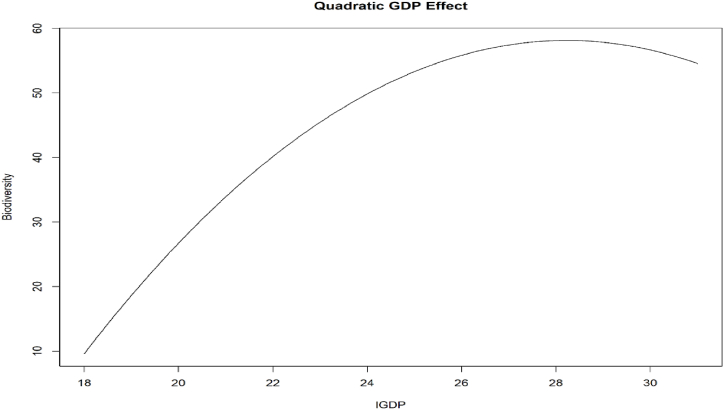
– Visualized quadratic effect of lGDP on Biodiversity.

Furthermore, this study interprets the PARDL model coefficients from Table 6. The long-run coefficients are estimated using equation (1), and the short-run are estimated using equation (2). The findings indicate that lGDP has a significant positive impact on biodiversity [5], while lGDP squared has a negative effect, constituting an inverted U-shaped impact [124]. Fig. 8 also provides visualized evidence of the inverted U-shaped effect. The Load Capacity Curve hypothesis supports this inverted U-shaped effect [125,126] (see Fig. 8).

The results further show that renewable energy consumption and financial technology (FinTech) significantly impact biodiversity in the long run. Studies like [98,127] have advocated for similar effects, arguing that both renewable energy and financial technologies can be leveraged to boost biodiversity in the long run, while their short-run effects are insignificant.

The results also showed that technological innovation and natural resource rent had a considerable negative influence on biodiversity, as demonstrated by Weiskopf et al. [128]. The scale effect, as measured by GDP, explains this phenomenon. Increases in both technology and natural resource use lead to increased growth and excessive resource use, which disturb the ecosystem.

The intercept negative value shows that all other excluded variables greatly restrict biodiversity in the economy, indicating an acceptable selection of independent variables with no excluded positive influence. A positive intercept would have omitted some unexplained positive effects, rendering them inefficient in enhancing biodiversity.

The overall convergence coefficient is negative and significant in the short-run, confirming that corrective changes in the dependent variable are associated with deviations from the long-run equilibrium. Thus, policymakers can intervene using the proposed independent variables to manage or boost biodiversity in the global economy.

**Fig. 9:**
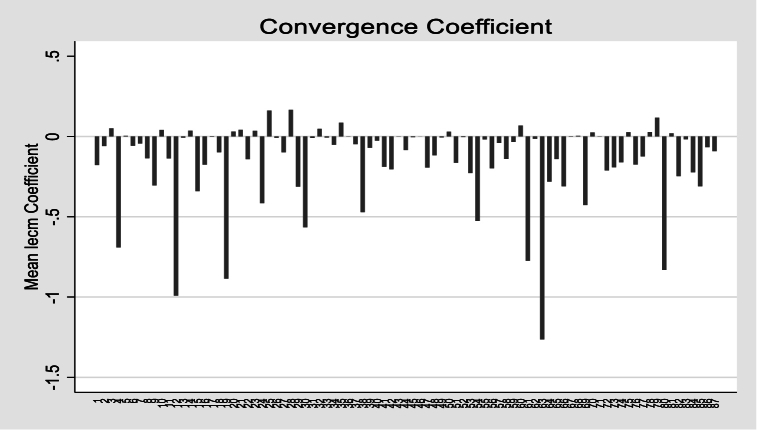
– Country-specific convergence coefficient.

The Fig. 9 illustrates the country-specific convergence coefficient using short-run estimates across cross-sections. The PMG model extracts these short-run estimates for each country. Except for a few countries where ECM is not negative, most countries converge to long-run equilibrium via negative ECM coefficients.

Table 7 provides the panel causality test, which examines causality for up to 2 lags. Here, we confirm causality for all independent variables running Westerlund towards biodiversity.

**Table 7:** – Panel Granger causality test.

Independent variable	Dependent Variable	Test	Probability
lGDP	BIOD	11.296	0.000∗∗∗
lIN	BIOD	7.104	0.000∗∗∗
REN	BIOD	9.793	0.000∗∗∗
NRR	BIOD	1.829	0.067∗
FINHET	BIOD	8.580	0.000∗∗∗

∗∗∗ significant at 1 % ∗ significant at 10 %.

## 5 Conclusion

### 5.1 Discussions

Human actions have had a profound impact on biodiversity around the world. There is an urgent need to develop techniques that enhance environmental and ecological well-being while striking a delicate balance between human and natural habitat requirements. This study used novel index-making methodologies and robust panel data analysis to investigate FinTech's impact on biodiversity. The data is collected from 87 countries, and the Yale University Environmental Performance Index is available between 2000 and 2022. The heterogeneous factor analysis took into account cross-sectional variations in the data for creating the FinTech index, which is based on financial development, mobile subscriptions, and internet access. The literature readily incorporates these FinTech objects. Furthermore, Pooled Mean Group (PMG) based Panel ARDL specification is conducted using Driscoll and Kraay (DK) estimates to make the model robust to cross-sectional dependence, while also addressing the issue of non-stationary variables and cross-sectional heteroscedasticity.

The results showed that increased economic activity had an inverted U-shaped effect, confirming the load capacity curve hypothesis (LCC). This demonstrated the need to curtail growth to a specific limit to prevent tradeoffs with biodiversity. While it suggests a limit on economic growth for sustainability, it also presents a viable alternative. It showed that growth can overwhelm the system, leading to ecosystem deterioration. Growing renewable energy can preserve biodiversity, while FinTech (FINHET) can make economic activity greener and more efficient, thereby improving biodiversity.

Another surprising outcome is that the natural resource rents, which harm biodiversity in the selected sample, are caused by innovation and limiting factors. Literature has pointed out natural resource extraction has environmental and climatic consequences [37]. Regulations must prioritize social gains over financial gains to curtail the scale effect of innovations, as they do not promote biodiversity.

### 5.2 Theoretical and econometric implications

First, this study highlighted that there is a relative coupling between economic activity and biodiversity, but it is the inverse of EKC and supports the LLC hypothesis. It encourages new research to find ways to decouple growth and biodiversity. Decoupling strategies may include circular economies, enhancement of sustainable agricultural practices, and conservation easements in addition to renewable energy, financial technology, and natural resource management. These strategies can encourage businesses to reduce their environmental impact while remaining profitable.

In terms of econometrics, this study introduced a factor analysis method better suited for creating indices from panel data. This method makes country-specific indices comparable across countries, as their mean and standard deviation are the same. Secondly, this study developed a modified form of the Panel ARDL—PMG model using its specification (functional form) in the Driscoll-Kraay estimator, thereby enhancing the model robustness to cross-sectional dependence. Future studies can extend these two novel approaches to apply them to different domains and assess their performance.

### 5.3 Practical implications

Our findings have significant practical policy implications for biodiversity conservation, economic growth, and technological innovation. This study discusses a strategy to curb biodiversity loss in 87 nations. Theoretically, this study develops a more robust and efficient way to find determinants of biodiversity, which is becoming an increasingly important policy goal around the world. Secondly, it examines the integration of financial development and technology in the form of FinTech, its far-reaching implications beyond growth, and its extensive effects.

The estimates offered policymakers various insights. The first consideration is the trade-off between economic growth and natural habitat protection. Growth strains natural habitats and biodiversity; therefore, growth targets must include sustainability. Decoupling economic growth and biodiversity may be possible by advancing renewable energy and FinTech. To achieve this equilibrium, green technology promotion and circular economy concepts must be implemented.

National policymakers should intervene in the innovation system to ensure that newly registered patents incorporate environmental, climatic, and biodiversity sustainability. Patent commercialization must prioritize long-term ecological outcomes. The government may subsidize social entrepreneurship and environmental patents to optimize benefits. Such regulations may include grants for startups focused on sustainable technologies or tax incentives for biodiversity-friendly firms.

Sustainable resource utilization and management are essential for ensuring that human needs for natural resources do not jeopardize natural habitats. Optimizing renewable resources and employing FinTech to oversee natural resource exploitation can ensure accountability for unsustainable activities.

Future studies can explore the scientific determinants of biodiversity documented at the national level to enhance the explainability of the model and assist policymakers in finding further solutions. Since the time period also includes COVID-19, future studies can include it as a variable to explore its implications.

## CRediT authorship contribution statement

**Shayan Khan Kakar:** Writing – review & editing, Writing – original draft, Software, Methodology, Formal analysis, Data curation, Conceptualization. **Jing Wang:** Writing – review & editing, Supervision, Funding acquisition. **Noman Arshed:** Writing – original draft, Software, Methodology, Investigation, Data curation. **Tran Thi Le Hien:** Writing – review & editing, Writing – original draft, Investigation, Data curation. **Nazir Muhammad Abdullahi:** Writing – review & editing, Writing – original draft, Formal analysis.

## Data availability

The data will be made available upon special request from the corresponding author.

## Declaration of competing interest

The authors declare that they have no known competing financial interests or personal relationships that could have appeared to influence the work reported in this paper.

## Figures and Tables

**Table:** – List of Countries

Algeria	Ecuador	Lebanon	Samoa
Antigua And Barbuda	Egypt, Arab Rep. Of	Libya	Saudi Arabia
Argentina	El Salvador	Malaysia	Seychelles
Aruba, Kingdom Of The Netherlands	Equatorial Guinea	Maldives	Singapore
Bahamas, The	Eswatini, Kingdom Of	Marshall Islands	Solomon Islands
Bahrain, Kingdom Of	Fiji, Rep. Of	Mauritius	South Africa
Barbados	Gabon	Mexico	Sri Lanka
Belize	Georgia	Micronesia	St. Kitts And Nevis
Bolivia	Ghana	Mongolia	St. Lucia
Botswana	Grenada	Morocco	St. Vincent And The Grenadines
Brazil	Guatemala	Namibia	Suriname
Brunei Darussalam	Guyana	Netherlands, The	Syrian Arab Rep.
Cabo Verde	Honduras	Nicaragua	Thailand
Cameroon	Hong Kong	Nigeria	Tonga
Chile	India	Oman	Trinidad And Tobago
China	Indonesia	Pakistan	Tunisia
Colombia	Iran, Islamic Rep. Of	Panama	Turkey
Congo, Dem. Rep.	Jamaica	Papua New Guinea	United Arab Emirates
Costa Rica	Jordan	Paraguay	United States
Côte D'Ivoire	Kenya	Peru	Uruguay
Dominica	Korea, Rep. Of	Philippines	Vietnam
Dominican Rep.	Kuwait	Qatar	

## Footnotes

1 The R squared does not provide a threshold to indicate model fitness. This low value indicates heterogeneity among the selected countries, which could be addressed in future studies using time series analysis.
